# Development of a psychosocial intervention to support informal caregivers of people with end-stage kidney disease receiving haemodialysis

**DOI:** 10.1186/s12882-020-02075-2

**Published:** 2020-10-01

**Authors:** Michael Matthews, Joanne Reid, Clare McKeaveney, Robert Mullan, Stephanie Bolton, Christopher Hill, Helen Noble

**Affiliations:** 1 School of Nursing and Midwifery, Queen’s University, Medical Biology Centre, 97 Lisburn Road, Belfast, BT9 7BL UK; 2 Renal Unit, Northern Health and Social Care Trust, 45 Bush Road, Antrim, BT41 2RL UK; 3 Regional Nephrology Unit, Belfast Health and Social Care Trust, 51 Lisburn Road, Belfast, BT9 7AB UK

**Keywords:** End-stage kidney disease, Informal carers, Psychosocial intervention, Supportive, Health care professionals, Systematic review, Medical research council framework

## Abstract

**Background:**

Patients with end-stage kidney disease, receiving haemodialysis rely increasingly on informal carers to help manage their debilitating chronic disease. Informal carers may experience a negative impact on their quality of life exacting a toll on their physical, social and emotional well-being. Informal carers of patients with end-stage kidney disease receiving haemodialysis have significant unmet needs which may include physical and psychological issues, financial disadvantage and social isolation. Poor experiences of informal carers may also impact the experience of the patients for whom they care. The needs of this group of informal caregivers have been largely neglected, with little emphasis placed on supportive interventions that might assist and support them in their caring role. The aim of this study is therefore to explore the experiences and unmet needs of informal carers of people with end-stage kidney disease receiving haemodialysis and develop a psychosocial intervention to support them in their caring role.

**Methods:**

This qualitative study will include a systematic review, semi-structured interviews with 30 informal carers and focus groups with renal health care professionals. Perceptions of care provision, caregiving experiences as well as contextual factors impacting the design and delivery of a psychosocial intervention for informal carers of patients with end-stage kidney disease, will be explored and will inform the development of a supportive intervention.

**Discussion:**

The needs of informal carers of patients with end-stage kidney disease have been neglected with little emphasis placed on supportive interventions that might assist and support this group in their care giving role. This is in contrast to other chronic disease groups such as stroke, cancer and dementia. In these conditions well developed supportive interventions have significantly improved outcomes in regard to informal caregivers’ preparedness, competence, positive emotions and psychological well-being in terms of informal care provision. Support interventions could potentially improve the quality of life of those informal carers who provide care to patients with end-stage kidney disease receiving haemodialysis.

## Background

There were 63,162 adult patients receiving renal replacement therapy in the United Kingdom (UK) in 2016 [1]. Up to 1.8 million people are being treated globally – 77% with chronic dialysis. Chronic kidney disease progresses along a five-stage trajectory, stage 5 is termed End Stage Kidney Disease (ESKD) [2] and occurs when the glomerular filtration rate of the kidney is less than 15 ml/min, thus requiring haemodialysis, peritoneal dialysis or transplantation [3]. Haemodialysis is a difficult and time-consuming treatment requiring patients to attend hospital three times a week for up to 4 h each time. During treatment, the patient is connected to a dialysis unit that filters their blood and removes waste products and excess fluid, replacing the role of the kidneys [2]. Despite technical advances and changes in the way haemodialysis is delivered, it remains a challenging, arduous treatment for many patients and impacts negatively on the lives of their informal carers [4].

Informal carers have been defined as any person, such as a family member, friend or neighbour who is giving regular, ongoing assistance to another person without payment for the care given [5]. Given the complexities of end-stage kidney disease and haemodialysis treatment and the profound effect they have on the patient and informal caregiver’s physical and psychological wellbeing, support for caregivers is vital [6, 7]. When an individual is affected with a chronic condition such as chronic kidney disease, life changes not only for the patient, but also for those who are emotionally and practically involved in providing care [4]. Functional dependence of patients often increases, leaving family members and friends to provide greater physical support. In addition to the provision of support in practical terms family members or friends are often required to provide emotional support and may have health and social care needs of their own that need to be addressed [8]. The impact of being an informal carer can be considerable [9] and is typically burdensome with informal caregivers often experiencing physical and psychological morbidity, financial disadvantage and social isolation [9]. Caregiving can extend for several years and may be equivalent to full-time employment [10]. Despite growing recognition of the burden and adverse effects on informal caregivers in the provision of care for individuals receiving dialysis there is a dearth of evidence about interventions that carers may find useful [11].

A systematic review found only three studies that evaluated interventions for caregivers of patients with end-stage kidney disease receiving haemodialysis [11]. These studies used only quantitative methods and included assessment of the effect of educational material on caregiver’s knowledge with no other outcomes being measured. There were no qualitative studies identified so there is poor understanding of the personal experiences of this group of informal carers who are likely to have unmet needs [5]. This systematic review highlights the current lack of evidence to help inform the development of supportive interventions for caregivers in this population [11]. A second review identified that further research is needed to explore the relationship between health services and renal carers in order to develop practical empowering interventions to assist them in their informal caring role [12]. Since the work completed by Tong et al. [11] and Low et al. [12] there has been no additional research carried out to develop supportive interventions for informal caregivers of patients with end-stage kidney disease receiving haemodialysis. In addition, there is no acknowledgement of the role of informal carers or their specific requirements in any of the guidance produced by National Institute for Health and Care Excellence (2019), National Service Framework (2004) or Renal Association Guidelines (2019).

There has been little acknowledgement of the needs of informal carers of end-stage kidney disease patients on haemodialysis. This is in contrast to other chronic illnesses such as stroke [13], cancer [14] and dementia [15]. In these conditions well developed interventions have included family training to support patients with rehabilitation, or to address unmet needs as a result of the patient’s illness. The Melbourne Family Support Programme is an example of one such intervention used in a palliative care setting. This programme involved the use of a psycho-educational intervention and produced significant improved outcomes in family caregivers’ preparedness, competence, positive emotions, levels of psychological wellbeing and unmet needs [9]. Enhanced psychosocial support can improve caregivers’ quality of life, satisfaction and coping ability and therefore enhance the care recipients overall medical and psychosocial outcomes [16]. With this in mind support interventions could potentially improve quality of life, satisfaction and ability to cope for those informal carers who provide care to end-stage kidney disease patients receiving haemodialysis. Many informal carers lack information about haemodialysis treatment and the expectations of their role, which can lead to worry and uncertainty [11]. They are often inadequately prepared, and the role is often unexpectedly put upon them [9].

## Methods/design

### Aim and objectives

The aim of this study is explore the experiences and unmet needs of informal carers of people with end-stage kidney disease receiving haemodialysis and develop a suitable psychosocial intervention to address their needs and support them in their caring role.

The objectives of this study are to -

- To complete a systematic review of previous research related to experiences, needs, and interventions developed for informal carers of patients with end-stage kidney disease receiving haemodialysis.
- To carry out semi-structured interviews with 30 informal carers to explore their experiences and needs and identify supportive interventions which may assist them in their informal caring role.
- Feedback findings from carer interviews to health-care professionals through focus groups in order to obtain their views on the potential development of a psychosocial intervention.
- To hold a national workshop in order to share findings from the semi-structured interviews completed with informal carers and focus groups completed with healthcare professionals and so inform the development of a psychosocial intervention for informal carers of patients with end-stage kidney disease receiving haemodialysis.

### Study population

This study will be carried out in two Renal Units within the United Kingdom. In combination these 2 units provides hospital-based haemodialysis for approximately 450 patients. The study protocol has been developed following the COREQ guidelines [17]. The Medical Research Council (MRC) Framework (2019) [18] for the Development and Evaluation of Complex Interventions will be used to guide the delivery of the study. This study aligns with the pre-clinical phase and phase 1 of the MRC Framework.

### Objective 1 – MRC framework – preclinical phase, review of the literature

This stage will include a systematic review [16] to identify needs and supports for informal carers of patients with end-stage kidney disease receiving haemodialysis including the content, organisation and delivery mechanisms of existing relevant interventions. This will identify gaps, current provision for informal carers in this population and inform study methods.

### Objective 2 – MRC framework – phase 1, interviews with informal carers

During this stage the researcher / corresponding author will explore the experiences, perceptions and opinions of informal carers of patients with end-stage kidney disease receiving haemodialysis. This information will be collected using semi-structured interviews. For the duration of the COVID-19 pandemic, the semi-structured interviews will be completed remotely using Skype or Telephone.

#### Recruitment and data collection

A purposive sampling technique will be used to recruit approximately 30 carers to take part in semi-structured interviews to explore the impact of informal caring. The figure of 30 participants was decided following review of sample size in previous qualitative research involving semi-structured interviews. It has been reported that 16–24 semi-structured interviews are needed to reach data saturation where a rich understanding of the phenomenon under study is gained [19]. In view of this it was felt a sample of 30 participants would be large enough given consideration to the scope of the study, complexity of the topic and the design of the study. Semi-structured interviews include open ended questions to address specific research questions whilst allowing the participant freedom of response [20]. Development of the interview schedule by the researcher / corresponding author and supervisory team is ongoing and is based on findings from the systematic review. The semi-structured interview will be piloted with 5 informal caregivers to elicit ease of understanding [21]. A topic guide will be prepared covering a list of issues to be covered with each participant. This flexible approach ensures the researcher elicits the information they require, whilst giving participants the opportunity to elaborate on issues which the researcher may not have considered [22]. Each informal carer will complete only one semi-structured interview. The number of carers approached, the number who agreed to participate in the study and the number of those who then declined to be interviewed for whatever reason will be captured and reported.

Initial contact with potential informal carers will be made by clinical gatekeepers who are senior nurses within the two haemodialysis units. Once potential informal caregivers are identified by the clinical gatekeepers, they will provide them with a covering letter detailing the main aim and objectives of the study and a copy of the patient information sheet, which they will give to the haemodialysis patient, so it can be provided to the informal caregiver. A contact number will also be provided to allow potential participants to contact the researcher if they have any queries or questions concerning any aspect of the study, and also to inform the researcher if they are willing to participate or not. A self-addressed envelope will also be provided which the informal carer may also return if they wish to participate in the study. The semi-structured interviews will be held at a mutually convenient time and place either in the informal carers own home or the dialysis unit. Written informed consent will be obtained before each interview. Posters outlining the study will be displayed within the haemodialysis unit and in the area where renal clinics are held in the outpatient department. Data collection will be completed when data saturation is reached, and no new information or themes are being uncovered [23]. A copy of the semi-structured interview guide for informal carers has been included as a supplementary file.

#### Recruitment of informal Carers

##### Inclusion criteria

- Aged 18 years or over
- Able to give informed consent
- Nominated by the patient as their main carer
- Caring for a patient living at home

##### Exclusion criteria

- Unable to give informed consent
- Not the primary carer
- Caring for a patient with a functioning kidney transplant
- Patients in inpatient setting

### Objective 3 – MRC framework – phase 1, focus groups with health care professionals

During this stage focus groups will be undertaken with renal healthcare professionals within the two Renal Units involved in the study. The researcher / corresponding author will feedback findings from the information obtained from the semi-structured interviews with informal carers on their personal experiences and thoughts on what would support them in their informal caring role. Key components for a psychosocial intervention and how this could be implemented in practice, will be explored and discussed based on the carer interviews. The outcomes from the focus group interviews will be used to inform the national workshop at the end of the study.

#### Recruitment and data collection

Purposive sampling will be used to recruit health care staff who are working in the renal speciality and are deemed to be expert in their specialist area through the positions they hold, these staff will be selected and invited to take part in the focus groups. Up to four focus groups with 6–8 participants per group will be undertaken. Collection of data will continue depending on availability of staff to participate. The optimum number to take part in a focus group is preferably 6 to 8 as it is felt to be a sufficient number to provide a variety of perspectives on a topic and small enough not to become disorganised and fragmented [20].

Recruitment to the focus group interviews will commence when the semi-structured interviews with carers have been carried out and data analysis completed. Initial contact with renal health care professionals will be made over a number of weeks during the clinical governance meetings which are held on a weekly basis and are attended by a wide range of renal health care professionals who have expertise in caring for haemodialysis patients and their carers. Updates on research taking place within the haemodialysis unit forms an integral part of these meetings. The researcher will present a brief overview on the progress of the study to date and highlight the need to recruit a group of renal health care professionals to take part in focus groups, and the rationale for this. The researcher will provide the renal health care professionals with a staff information sheet and they will be requested to self-refer into the study through making e-mail or telephone contact with the researcher. Posters will also be displayed within the haemodialysis unit advertising the study. When health care professionals have verbally consented to take part in the focus group interviews, the researcher will arrange a suitable time and place for the focus group interviews to take place, it is most likely the focus groups will be held in the renal unit where the healthcare professionals are based. Prior to commencing the focus groups, written informed consent will be obtained from each participant. For the duration of the COVID-19 pandemic, the focus groups will be completed using Skype or Microsoft Teams. A copy of the focus group interview schedule has been included as a supplementary file.

#### Recruitment of health care professionals

##### Inclusion criteria

- Presently working for at least 2 years within the renal speciality and have a permanent contract with either the Northern Health and Social Care Trust or the Belfast Health and Social Care Trust.
- Have an appropriate professional qualification (ie - Registered General Nurse, Doctor of Medicine) in their field of work

##### Exclusion criteria

- Health care professionals not involved in care delivery to patients with ESKD on haemodialysis and their carers
- Health care professionals who are employed by a recruitment agency or do not hold a permanent contract within the Health and Social Care Trust in which they are employed

### Objective 4 – MRC framework - phase 1, supportive intervention components

Following stage 2 and 3, the researcher will work with informal carers, patients, staff working within the renal unit, policy makers and other key stakeholders such as Northern Ireland Kidney Patients Association (NIKPA). A national workshop will be held within one of the Hospitals where the research took place and will be facilitated by representatives from Kidney Care UK and the Renal Association. The findings of the study will be presented at the workshop which will explore and discuss the potential key components of a psychosocial intervention for informal renal carers taking into account the information obtained from the semi-structured and focus group interviews. Breakout sessions with feedback will be facilitated where delegates will be split into groups and asked to explore how carers can be best supported and to comment on the proposed psychosocial intervention. As well as impacting service development at a local level, the national workshop has also the potential to impact services nationally, as this research will be disseminated through publications and presentations made at conferences.

### Theoretical framework

The main focus of the intervention will be to enable informal carers to cope with the holistic stressors resulting from their caring role. It will be informed by the Transactional Model of Stress and Coping [24]. This model proposes that it is not the informal caring role itself that is stressful, but rather the carer’s appraisal of the situation and judgement of their behaviour in relation to how they cope. The proposed components of the intervention will take into account the stressors and coping mechanisms identified by informal carers and aim to facilitate effective coping, preparedness and competency to ultimately improve both the quality of life of the informal carer and the patient on haemodialysis for whom they provide informal care.

### Data analysis

The data from the recorded semi-structured interviews and focus groups will be transcribed verbatim, checked for accuracy against the original audio files and read several times [20]. Field notes will also be made during the semi-structured interviews and focus groups. A thematic content analysis will be carried out using a method outlined by King and Horrocks [25]. This approach has been chosen as it aligns with the interpretivist approach of the study. The researcher is aiming to interpret and understand informal carers experiences of caregiving to patients with end stage kidney disease receiving haemodialysis and the views of healthcare staff. Thematic analysis is a flexible and useful research tool, which provides a rich and detailed, complex account of data collected [26]. During the process of thematic analysis, the researcher / corresponding author will analyse the data to identify common themes and group recurring themes occurring in the data. This will incorporate coding of data via open coding to organise and assimilate the raw data. This will provide an insight and assist in gaining an understanding into the complex views, opinions and experiences of the informal carers. In the focus groups themes related to intervention development and implementation will be identified. Data management will be assisted by using NVivo version 11 (QSR 2015) [27].

### Study withdrawal

***Participants*** will be advised of the voluntary nature of their inclusion in this research and the informal carers and health care professionals can withdraw from the semi-structured interviews and focus groups at their request. As sensitive issues may be identified and discussed during the semi-structured interview and focus groups potential respondents may become distressed. A distress protocol will therefore be developed for use within the study. Additionally, as it is anticipated that some interviews may be conducted in informal carers homes, a lone worker policy will be used within the study. As stated within the information sheet, for any informal carer or health care professional who does withdraw, we will use collected data up to the withdrawal point and this data may be used in the data analysis plan. We will record all reasons for withdrawal during the data collection of this study.

### Patient and public involvement (PPI)

This proposal has been developed in collaboration with members of NIKPA, informal caregivers and health care professionals who have contributed to the study proposal and have been influential in the shaping and refining of the final proposal. Following a recent consultation in August 2019 with four informal caregivers’ they agreed that the research topic is very relevant and warrants further exploration. Informal carers identified little support for performing their caring duties. NIKPA have been consulted on the study proposal, and helped in redefining the lay summary and aims and objectives. NIKPA strongly advocated the need for a collaborative approach with informal carers at all stages of the research to ensure the project design is enhanced by their experiences. They have agreed to provide advice through quarterly meetings and will be central to the dissemination of the findings of the study. Personal and public involvement will be incorporated in the reporting and dissemination of the study through a variety of different mediums. Information will be shared by attending meetings of the NIKPA and the Renal Support Group in the haemodialysis units, where renal patients and their family members meet every 3 months to share experiences. Patients and carers will be involved in the dissemination of user friendly reports, newsletters and involvement in conference presentations and publications arising from the study.

An advisory group will be established to support the study and will include service users, healthcare staff, policy makers, key stakeholders and academics from the academic institution in which the researcher is based. The advisory group, who will meet regularly, will provide expertise and a strategic view on the development of the research study. They will assist with all stages of the research project from the development of the research design through to dissemination of the research study findings.

### Ethical considerations

The study will be conducted in accordance with the principles of the International Conference on Harmonization Good Clinical Practice Guidelines [28] (ICH-GCP). Sponsorship for the study will be obtained from Queen’s University, Belfast the academic institution where the researcher is based. Ethical approval has also been gained from the West of Scotland Research Ethics Service – REC reference: 20/WS/0056. In addition, governance approval will be obtained from the two Research and Governance Offices in both Health and Social Care Trusts where the research is being carried out. Data will be retained in accordance with General Data Protection Regulations (GDPR) (2018). Fundamental principles of good practice including the provision of user friendly information sheets, informed consent, voluntary participation and the opportunity to withdraw from the study at any time, confidentiality and general data protection procedures will be applied as a minimum standard. All members of the research team will be required to have completed Good Clinical Practice (GCP) training and the researcher/corresponding author has experience in carrying out healthcare research and has previously worked as a clinical research nurse. The study has been peer reviewed by the clinical lead for Nephrology in both of the Health and Social Care Trusts where the research will be carried out.

## Discussion

This is the first study that will result in the development of the components of a supportive intervention to support informal carers of patients with ESKD receiving haemodialysis by engaging with and working in collaboration with informal carers and health care professionals.

The support offered by informal carers who are generally family members, friends or neighbours, is an important role in helping patients with end-stage kidney disease, receiving renal replacement therapy to adjust to the illness and to increase adherence to treatment regimens [12]. The provision of support from informal carers is a complex role which can have a negative effect on the caregiver’s physical and psychological wellbeing [14]. It has been reported that informal caregiver burden in patients with end-stage kidney disease receiving haemodialysis was associated with less social support and lower health related quality of life for both informal carers and the patients for whom they are providing care [16]. Evidence indicates that regardless of country where the haemodialysis setting is based informal caregivers experience a high level of burden [28]. The informal caregiver has to be involved in assisting with daily living activities, which may include hygiene, providing meals and financial resources, as well as potentially dealing with other chronic illnesses, such as stroke and dementia, which further highlights the complexity of this role [13, 15].

In the systematic review completed by Tong et al. [11] the three studies identified that evaluated an intervention for caregivers of chronic kidney disease patients, assessed only the effect of educational material on informal caregivers’ knowledge. Two evaluated information provided to caregivers of dialysis patients using a pre and post-test study design. The other study used participatory action research methods to develop and evaluate an information handbook for transplant patients and their caregivers. No other outcomes were reported.

From this research we will identify the experiences and unmet needs of informal carers of people with end-stage kidney disease receiving haemodialysis and how they can be best supported in their care giving role and the subsequent development of a psychosocial intervention to support this group of carers. The development of a psychosocial intervention will allow future research to be designed which aims to test this intervention which will be aimed at improving caregivers’ quality of life, satisfaction and coping ability and therefore enhance the care recipients overall medical and social outcomes [16]. Qualitative study designs are needed to elicit a deeper understanding of the informal caring role, the caregiver-patient relationship, and the psychological aspects of informal caring [29]. This will subsequently lead to the development of effective support mechanisms to assist in the caregiving role.

## Conclusion

In other chronic disease groups well developed interventions have been shown to improve caregivers’ quality of life [9, 13]. Despite informal caregiver burden being a focus of research in the context of haemodialysis there is a lack of developed and tested interventions to support informal carers of patients with end-stage kidney disease undergoing haemodialysis. There is emerging evidence that suggests that maintaining the physical and psychological well-being of informal carers, enables them to care effectively, which in turn will also improve the experience of the person being cared for. Future research is therefore required to understand the experiences and needs of this group of informal carers, so that supportive interventions can be developed.

## Supplementary information

**Additional file 1.** Semi-structured interview schedule for informal carers.

**Additional file 2.** Focus Group interview schedule for healthcare professionals.

## Data Availability

The dataset used and or analysed during this study will be available from the corresponding author on request.

## Abbreviations

UK: United Kingdom
ESKD: End-Stage Kidney Disease
MRC: Medical Research Council
NIKPA: Northern Ireland Kidney Patient Association
ICH-GCP: International Conference on Harminisation Good Clinical Practice Guidelines
ORECNI: Office for Research Ethics Committees, Northern Ireland
GDPR: General Data Protection Regulations
GCP: Good Clinical Practice Guidelines
NIKRF: Northern Ireland Kidney Research Fund
