# Supplementary material for: Development of a psychosocial intervention to support informal caregivers of people with end-stage kidney disease receiving haemodialysis
Source: BMC Nephrol. 2020 Oct 1;21:421. doi: 10.1186/s12882-020-02075-2 (PMC7527291; doi:10.1186/s12882-020-02075-2)
Supplement: Supplementary file 1 — Additional file 1. Semi-structured interview schedule for informal carers. [file 12882_2020_2075_MOESM1_ESM.docx]

**Semi-structured Interview schedule for Informal Carers**

Thank you for taking part in this semi-structured interview, the purpose of this interview is to learn about your experiences and opinions of your informal caring role and to identify if there is anything you would find beneficial and might help you in your role. This would lead to the development of a psychosocial intervention to support you in your informal caring role. What is discussed during this interview is confidential and anonymity will be maintained. The interview will be audio-recorded and transcribed.

**Demographics**

Including name, age, marital status, duration of informal caring role, do you live with the person with whom you are caring, relationship to the person for whom you are providing care, hospital in which your relative/friend receives dialysis.

**Open Invitation Statement**

Tell me about your caring role at present?

**Prompts**

- Who you are providing care for
- How long you have been in this caring role
- Other previous/current experiences of caregiving
- Do you live with the person for whom you are providing informal care?

**Feelings and Emotions**

How does informal caring impact your life and daily activities?

**Prompts**

- Can you tell me about your experiences of your informal caring role?
- Can you tell me what feelings you experience day to day as an informal carer?
- What has the emotional impact been of being an informal carer?
- Can you tell me what emotional experiences have surprised you/are new to you?
- Can you highlight any positives that may have come out of your caring role?
- If there are any challenges/negative experiences, is there anything that would help you cope
- Is there anyone you can feel you can speak with if you are experiencing particular challenges or difficulties during your informal caring role

**Social Factors**

Can you describe to me how your social life has changed since starting your caring role?

**Prompts**

- How has life impacted on relationships with your children/partner/siblings/relatives or close friends since becoming an informal carer?
- Can you describe social benefits you may have experienced?
- Can you describe any challenges you may have experienced in your social life since becoming an informal carer?

**Physical Factors**

Can you tell me about the impact the informal caring role has had on your physical health?

**Prompts**

- Can you tell me about the physical requirements of your caring role?
- What has been the impact on your body?
- Looking back at the duration of your caregiving role, have you seen a change in the physical needs of the person for whom you are caring?

**Financial Implications**

Can you tell me about the financial implications of your caring role?

**Prompts**

Tell me about the financial practicalities you experience in carrying out your informal caring role in terms of eg - childcare, housing, insurance, transport

Could you tell me about any supports and or barriers you have faced relating to these aspects when carrying out your informal caring role.

**Current/future support mechanisms**

Do you feel you have received enough support to assist you in your informal caring role?

**Prompts**

- What support have you received from healthcare professionals during this current caregiving experience
- What support have you received from family members/friends during this current caregiving experience
- Do you think healthcare providers have enough insight into your caring role to offer necessary support?
- Do you think family members/friends have enough insight into your caring role to offer necessary support?
- Do healthcare providers or any other family member/friend motivate or encourage you to use self-management strategies to reduce or assist in managing challenging circumstances?
- What support would you find most beneficial to assist with you in your informal caring role?
- Can you describe to me what coping skills you have used in your informal caring role?
