# Supplementary material for: Development of a psychosocial intervention to support informal caregivers of people with end-stage kidney disease receiving haemodialysis
Source: BMC Nephrol. 2020 Oct 1;21:421. doi: 10.1186/s12882-020-02075-2 (PMC7527291; doi:10.1186/s12882-020-02075-2)
Supplement: Supplementary file 2 — Additional file 2. Focus Group interview schedule for healthcare professionals. [file 12882_2020_2075_MOESM2_ESM.docx]

**Focus Group Interview schedule for Healthcare Professionals**

Thank you for taking part in this focus group interview, the purpose of this focus group is to is to feedback findings obtained from the semi-structured interviews with informal carers on their personal experiences and thoughts on what would support them in their informal caring role. Key components for a psychosocial intervention and how this could be implemented in practice, will be explored and discussed. The information provided and discussed during this focus group interview is confidential with the exception that if information is disclosed that indicates that an individual is at risk of harming themselves or others, or in danger of being harmed by someone else, the researcher is legally obliged to pass on this information to appropriate personnel. Anything raised during this interview should not be discussed outside this room. Information provided during this focus group interview may be published as a report, confidentiality and anonymity will be maintained so that no person can be identified from publications. This interview will be audio-recorded so that it can be transcribed verbatim and checked for accuracy when analysing the data.

**Demographics**

Including profession, years of experience in renal setting, hospital in which you are based.

The format of this focus group interview will be based on the five core areas discussed with the informal carers during their semi-structured interviews which included –

1. Feelings and Emotions
2. Social Factors
3. Physical Factors
4. Financial Implications
5. Current/future support mechanisms.
